# The structural deficit of the Olympics and the World Cup: Comparing costs against revenues over time

**DOI:** 10.1177/0308518X221098741

**Published:** 2022-05-31

**Authors:** Martin Müller, David Gogishvili, Sven Daniel Wolfe

**Affiliations:** Department of Geography and Sustainability, 27213 University of Lausanne, Lausanne, Switzerland; Department of Geography and Sustainability, 27213 University of Lausanne, Lausanne, Switzerland; Department of Geography and Sustainability, 27213 University of Lausanne, Lausanne, Switzerland

**Keywords:** Cost, revenue, mega-events, Olympic Games, Football World Cup

## Abstract

The Olympic Games and the Football World Cups are among the most expensive projects in the world. While available theoretical explanations suggest that the revenues of mega-events are overestimated and the costs underestimated, there is no comprehensive empirical study on whether costs exceed revenues. Based on a custom-built database from public sources, this article compares the revenues and costs of the Olympic Games and World Cups between 1964 and 2018 (*N* = 43), together totalling close to USD 70 billion in revenues and more than USD 120 billion in costs. It finds that costs exceeded revenues in most cases: more than four out of five Olympics and World Cups ran a deficit. The average return-on-investment for an event was negative (– 38%), with mean costs of USD 2.8 billion exceeding mean revenues of USD 1.7 billion per event. The 1976 Summer Olympics in Montréal, the 2014 Winter Olympics in Sochi and the 2002 World Cup in Japan/South Korea recorded the highest absolute deficits. The Summer Olympics 1984 in Los Angeles, the Winter Olympics 2010 in Vancouver and the 2018 World Cup in Russia are among the few events that posted a surplus. The article concludes that the Olympic Games and the Football World Cup suffer from a structural deficit and could not exist without external subsidies. This finding urges a re-evaluation of these events as loss-making ventures that lack financial sustainability.

## Introduction

This paper answers a seemingly simple question: Are mega-events profitable? In other words, are their financial revenues greater than their costs? The answer to that question has remained elusive, mostly because revenues and costs accrue to different organisations, which, in addition, are not very transparent or coherent about those figures. Are the Olympics and the Football World Cup profitable for the International Olympic Committee (IOC) and the Fédération Internationale de Football Association (FIFA), who own the rights to these events? Yes, very much so. Are they profitable for the organising committees that need to put them on? Sometimes, but not very often. For the host city and government? Hardly ever.

The high costs of mega-events are well-established (e.g. Baade and Matheson, 2016; Flyvbjerg et al., 2021; Zimbalist, 2015) as are their problematic effects on host cities (e.g. Boykoff, 2014; Müller, 2015b). We do not know, by contrast, whether overall revenues might exceed costs. Supporters of the Olympics maintain that costs may be high but revenues are even higher (see Serhan, 2021). If the opposite was true, however, it would mean that these events run a structural deficit and are unable to pay their own way, even if the distribution of revenues was less skewed in favour of the IOC and FIFA as it is now. Theoretical considerations, drawn from principal-agent theory, auction theory and behavioural economics, would suggest the presence of systematic underestimation of costs and overpromising of benefits.

The question about the profit or deficit of mega-events is crucial for citizens and decision-makers in times when hosting mega-events is becoming more and more contested. Boosters continue to praise events’ transformative capacities as catalysts for urban and regional development and seek to mobilise ‘best practices’ from around the world for hosting (see Lauermann, 2014; Trubina, 2019; Silvestre, 2020; Temenos and McCann, 2012 for critical analyses). At the same time, an increasing number of cities and countries experience more and more opposition to hosting these events, leading to negative referendum outcomes and shelved bids (Boykoff and Gaffney, 2020; Kassens-Noor, 2019; Lauermann and Vogelpohl, 2019).

This paper seeks to establish the profitability (or not) of mega-events by presenting an event-level comparison of the major financial revenues and costs of the Summer and Winter Olympics and the Football World Cup from the 1960s to the late 2010s. In so doing, it advances existing scholarship that has tended to focus on one of the two events (e.g. Baade and Matheson, 2016; Essex and Chalkley, 2004 on the Olympics; Fett 2020 on the World Cup), on one organisation (organising committee, FIFA/IOC, government), on either costs or revenues (e.g. Flyvbjerg et al., 2021 for costs) or on a rather short time period (e.g. Graeff and Knijnik, 2021 on the period from 2006 to 2022). Such a systematic, longitudinal comparison is important, because it allows identifying larger patterns across events and over time, and it avoids generalising from a small sample of just one, or few, events. It also makes it possible to discern differences between the three events, establishing which might be more and which less profitable.

Taking three of the largest mega-events – the Olympic Summer Games, the Olympic Winter Games and the Men’s Football World Cup – the paper analyses longitudinal data on three major revenue streams (broadcasting, sponsorship and tickets) and two major sources of cost (organisation and sports venues), covering the 43 editions of these events between 1964 and 2018, with a total of more than USD_2018_ 120 billion in costs and more than USD_2018_ 70 billion in revenues (which is about seven times the annual operating budget of the City of Los Angeles, to provide a measuring rod (City of Los Angeles, 2021)). It examines the unit revenue and unit cost of these events by relating revenues and costs to the number of athletes and tickets at these events. It finally plots the 43 events in a matrix, according to their cost and return-on-investment, distinguishing them according to their overall cost and their relative profitability.

## Literature review

“Will it be worth it?” This question has vexed policy-makers, citizens and scholars confronting mega-events. Given the enormous costs associated with these events, nowadays often in excess of USD 10 billion, this question is of great relevance, in particular given the share of public funding ploughed into many mega-events. The economic dimensions of mega-events have been researched in two major ways. First, literature on economic *impacts* has examined to what degree the expenditure related to mega-events has had an effect on the economy of a region or a country. These impacts are typically expressed as a change in GDP, income, employment, tax receipts and other economic indicators. The literature here is large and varied and a general consensus has emerged that ex-ante studies tend to overestimate impacts and that the typically small (or even negative) economic impacts do not justify the outlays connected to mega-events (e.g. Baade and Matheson, 2016; Coates and Humphreys, 2008; Porter and Fletcher, 2008; Zimbalist, 2015; see Scandizzo and Pierleoni, 2018 for a comprehensive survey).

**Figure 1.**
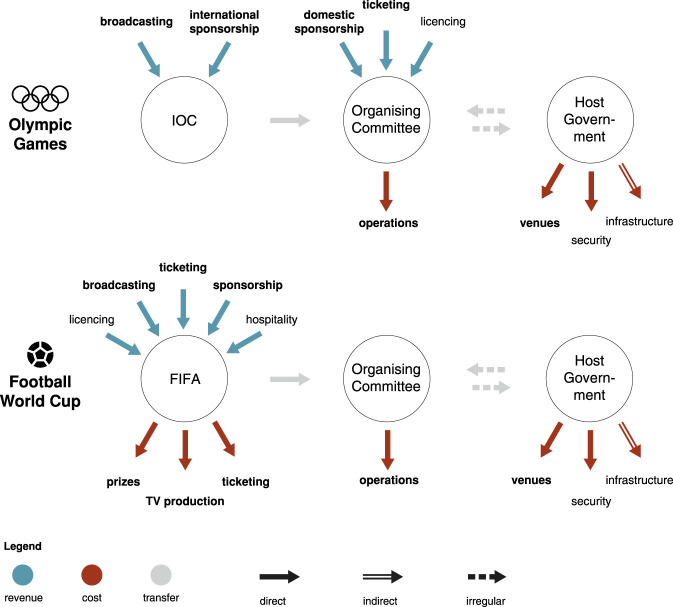
Cost and revenue streams in the Olympic Games and the Football World Cup. Costs and revenue streams covered in this article are in bold.

The second body of research has examined the *revenues and costs* of mega-events. These refer to income and expenditure that show up on the balance sheet of an organisation involved in organising those events, as illustrated in Figure 1. This may be the IOC or FIFA, the organising committee or various departments of the host city, region or country. One can distinguish between direct revenues and costs, which can be immediately attributed to staging the event itself, such as operations and event venues, and *indirect* revenues and costs, which support the staging of the event without being essential to it (Flyvbjerg, Budzier et al., 2021). Indirect costs are typically those related to general infrastructures, such as transport, accommodation, and others, which may or may not have been occasioned by the mega-event and whose utility is not limited primarily to the event itself. Thus, an airport expansion in preparation for hosting the World Cup may have been occasioned by the World Cup, but it can serve the region long after the World Cup is gone.

Figure 1 shows the distribution of those financial flows for the Olympic Games and the World Cup. It becomes immediately clear that the World Cup is much more centralised, with all of the revenues accruing to FIFA and most of the operational costs borne by FIFA, either directly or via a transfer to the Organising Committee, which usually covers the full operational costs (Zimbalist, 2015: 34). The Olympic Games, by contrast, have revenues accruing both to the IOC and the Organising Committee, while costs are incurred by the Organising Committee and the host government. The various levels of host government (city, region, nation) have no direct revenues for both the Olympic Games and the World Cup but incur most of the costs. The distribution of costs and revenues in Figure 1 underscores the frequent critique that the costs of mega-events are socialised, while the profit is privatised (Boykoff, 2014).

Previous research on the revenues and costs of mega-events is plentiful, but studies tend to focus on individual cases. By contrast, research with a longitudinal approach comparing several event types (Summer Olympics, Winter Olympics, World Cup etc.) is rare. Table 1 lists the studies that compare revenues or costs for more than five event editions. Of these, only one (Graeff and Knijnik, 2021) compares revenues and costs across the Olympic Games and the World Cup, though in a small sample (five World Cups, three Summer Olympics). It finds that the costs of the event consistently exceed the revenue. Two other studies (Baade and Matheson 2016; Matheson 2018) report similar findings for the Olympic Games and the World Cup separately, using somewhat larger samples.

**Table 1.** Studies on revenues and costs of the Olympic Games and the Football World Cup with *N* > 5.

		Event			Finance		Cases
Year	Source	Summer Olympics	Winter Olympics	World Cup	Revenue	Cost	
2002	Chappelet		×		×	×	Lake Placid 1980 – Salt Lake 2002
2004	Essex and Chalkeley		×		×	×	Chamonix 1924 – Turin 2006
2004	Preuss	×			×	×	Munich 1972 – Beijing 2008
2016	Baade and Matheson	×	×		×	×	Seoul 1988 – Rio 2016 Nagano 1998 – Sochi 2014
2018	Matheson			×	×	×	USA 1994 – Qatar 2022
2019	Preuß et al.	×	×		×	×	Sydney 2000 – Rio 2016 Salt Lake 2002 – PyeongChang 2018
2020	Fett			×	×	×	Brazil 1950 – Russia 2018
2021	Flyvbjerg et al.	×	×			x	Rome 1960 – Rio 2016 Squaw Valley 1960 – Sochi 2014
2021	Graeff and Knijnik	×		×	×	×	Beijing 2008 – Rio 2016 Germany 2006 – Qatar 2022
	This study	×	×	×	×	×	Tokyo 1964 – Rio 2016 Innsbruck 1964 – PyeongChang 2018 England 1966 – Russia 2018

Several other studies in Table 1 focus on a selection of revenues and costs. Preuß et al. (2019) study the budgets of the organising committees of recent Summer and Winter Olympics and find that revenues cover expenditure. Yet, they only present a restricted analysis of revenues and costs beyond the organising committee. Flyvbjerg et al. (2021)’s is the most systematic analysis of costs and cost overruns, establishing that every Olympic Games has suffered from a cost overrun and that average cost overruns are larger than with other mega-projects (but see Preuss, 2022). For the World Cups, Fett’s (2020) examination is the most comprehensive, but he only considers broadcasting rights on the revenue side.

Some more dated studies also review revenues and costs, without comparing them directly, however. Preuss (2004) is the most detailed of this lot, but only considers the Summer Games. Analysing the budgets of the organising committees, he finds that these tend to end up with an operational surplus. Chappelet (2002) and Essex and Chalkley (2004) undertake similar studies for the Winter Games, but with a focus only on broadcasting revenues (Essex and Chalkley), or broadcasting and sponsorship revenues (Chappelet), excluding ticket sales.

These studies demonstrate that the Olympic Games and the World Cup are lucrative for the organisations that own them – the IOC and FIFA. The organising committees often manage to break even (Preuss, 2004; Preuß et al., 2019), but may require additional subsidies from the government or the rightsowners to do so. For cities and regions, by contrast, mega-events are a losing game in most cases: ‘the Olympic Games as currently conducted are not economically viable for most cities’ (Baade and Matheson, 2016: 214).

Our study extends this existing research with a systematic comparison of the major costs and revenues of the Olympics and the World Cup by analysing time-series data covering the period from the 1960s to the 2010s. As such, it allows establishing whether these events overall have a surplus or a deficit, and examining the possibility of generalization of the findings of previous studies. It also makes it possible to identify potential differences between the Summer Olympics, the Winter Olympics and the World Cup and how profit or loss evolve over time. Finally, our analysis allows testing theoretical assumptions, which predict a systematic overestimation of benefits and underestimation of costs for mega-events, as we explain in the following.

### Theoretical considerations

Previous research has sought to advance theoretical explanations for better understanding revenue and cost dynamics in the Olympic Games. Such explanations draw on diverse theories, most importantly principal-agent theory, auction theory and the theory of cognitive biases, which have become a cornerstone of behavioural economics. Like any other complex and costly project with multiple stakeholders, mega-events are subject to optimism bias and strategic misrepresentation. *Optimism bias* is a cognitive bias that leads to the adoption of unrealistic, overly optimistic assumptions regarding project outcomes (Lovallo and Kahneman, 2003). In this case, it results in the well-known symptom of overpromising benefits and revenues, while underestimating costs and time of completion for mega-events (Whitson and Horne, 2006). *Strategic misrepresentation* is the deliberate manipulation of cost and revenue forecasts to create a more favourable impression of a project, often by, again, lowballing costs and exaggerating benefits (Flyvbjerg et al., 2003). This is possible because of a principal-agent situation and information asymmetry, in which the agent (for example the city bidding for a mega-event) knows more about the real costs of a mega-event than the principal (the taxpayers), but communicates a lower cost estimate to make hosting the event more palatable to the public (Preuß et al., 2019).

This general problematic is compounded by several circumstances specific to mega-events. Flyvbjerg et al. (2021) identify constraints that all lead to escalating costs. For one thing, the decision to host a mega-event, once the right has been won, is *irreversible*. It is, therefore, necessary to see it through, even when more precise forecasts of benefits and costs, as they often become available after the bid, lead to more negative results. What contributes to escalating costs is the *impossibility of a schedule-budget trade-off*. Unlike with most other mega-projects, the deadline for delivering a mega-event is set to the minute, several years in advance. The only way to compensate for delays in preparation is therefore to throw more money at the preparation work. The full deficit guarantee, which is often required from host countries, covers these expenses unconditionally, leading to an incentive to further overspend. Finally, hosts suffer from *eternal beginner syndrome*, as the rotation of mega-events worldwide ensures that most people delivering the event in the host location lack any experience of the specificities and complexities of this project.

In addition, Preuß et al. (2019) highlight several other dynamics amplifying cost overruns in the Olympic Games. While they are subject to a *cascading chain of principal–agent relationships* that amplify the potential misrepresentation of costs and benefits, the uneven distribution of costs and revenues between rightsowners and hosts additionally leads to a *moral hazard* for the IOC and FIFA: as they define the requirements of the event that the host must meet but only incur a small part (or none) of the costs, there is an incentive to set an excessive scope of requirements, leading to ballooning costs. Furthermore, as up until today most mega-events have been awarded through an auction system of bidding, they have been afflicted by what is known as the *winner’s curse*. This idea, developed from auction theory, describes a situation where several parties are bidding at an auction for an object with an uncertain value (in this case cities and countries bidding for the right to host the Olympic Games or the Football World Cup). The highest bid will likely come from the city and country that overestimates the true value of the mega-event, and therefore will end up paying more than it will receive in benefits.

### Research design

To establish whether the costs of mega-events systematically exceed the revenues, and therefore to confirm or refute the theoretical assumptions, our research design includes the major sources of revenues and costs. In addition, we compiled a longitudinal sample to be able to trace the evolution of revenues and costs over time. Finally, we included several event types (Summer Games, Winter Games, World Cups) to examine whether there exist differences between different types.

#### Sources of revenue and cost

We included three types of revenue:

- Revenue from broadcasting rights
- Revenue from (domestic and international) sponsorship
- Revenue from ticket sales

Previous research has found that these three revenue sources account for more than 90% of total revenues in recent Olympic Games and World Cups (Baade and Matheson, 2016: 206; Matheson, 2018: 23) and therefore allow a robust approximation of revenues.

For delimiting the costs, we followed Flyvbjerg et al. (2021) by including the following two types of costs:

- Operational costs
- Sports venue costs

Similar to Flyvbjerg et al. (2021) and Preuß et al. (2019), we did not include indirect costs, such as for the expansion of hotel capacities, new public transport, airport extensions, improvements to power supply etc. Their overall amount often exceeds that of direct costs. We do not include these indirect costs in our scope, because the event-induced share of those costs is hard to delimit (Baade and Matheson, 2016) and it is difficult to judge to what extent these costs occurred because of the event or would have been occurred in any case (cf. Kassens-Noor, 2012). We also did not include costs for the Olympic village, media centres etc., as these are not directly necessary for the event. Typically, these range between USD 0.1 and USD 1 billion (see Preuß et al., 2019 for the most systematic comparison). Bidding costs, although often neglected (de Nooij, 2014), are also outside our scope, as these are poorly documented and typically small compared to other costs. Finally, we also did not include in-kind costs, such as government secondment and security (for which accounting standards vary greatly) and opportunity costs (which are specific to each context and hard to quantify). The exclusion of indirect costs and in-kind costs makes our cost estimate conservative, that is, we tend to underestimate costs and therefore overestimate any potential profitability.

#### Data collection and processing

Data were collected from a number of publicly available sources, including official reports from organising committees, the IOC, FIFA and host governments and occasionally audit reports, media and the academic literature, where the aforementioned sources did not yield any conclusive data (see Müller et al., 2021 for a detailed description of our approach). Eight out of a total of 215 data points, equivalent to 3.7%, are missing. To draw meaningful comparisons between the various currencies from different time periods, we first converted values to US-Dollars (USD) using the World Bank national currency unit values and then applied the World Bank Consumer Price Index to inflate to the base year of 2018 (see Turner et al., 2019 for the method adopted; and Essex and Chalkley, 2004 for a similar approach when comparing Olympic costs). We thus arrive at USD_2018_, which allows comparing monetary values in real terms, adjusted for inflation. Since the IOC’s global sponsorship programme does not break down revenue per Olympic Games (only per quadrennium), we applied a 2:1 split between Summer and Winter Games. We calculated a volatility index for indicators by dividing the standard deviation by the mean (resulting in what is known as the coefficient of variation). The dataset is available on Harvard Dataverse (Müller et al., 2022).

#### Sample delimitation

We chose to include the Summer Olympic Games, the Winter Olympic Games and the Football World Cup in our sample, as these are among the largest events in the world (Müller, 2015a). The comparison between a single sports event in multiple locations (the World Cup) and a multi-sports event in one location (the Olympic Games) allows us to see differences between event types. Extending from 1964 to 2018, our sample includes 14 Summer Olympic Games, 15 Winter Olympic Games and 14 World Cups and thus a total of 43 events. Our sample starts in 1964 for two reasons. First, the early 1960s mark the beginning of a period of strong expansion in the size of these events, with the development of live satellite transmission and the increasing impact of urban interventions linked to these events (Essex and Chalkley, 1998; Horne and Whannel, 2020). Second, data availability for our revenue and cost streams is limited before 1964, so any extension to before that date would increase the number of missing values significantly and make our analysis less robust

#### Limitations

Our research design presents a number of limitations. First, while we include the most important items of costs and revenues, we do not cover all. Our calculations are therefore best understood as approximations, based on the available data. Second, investment in sports venues can be written off over a longer period and one might argue that the cost should not be solely imputed to the event that occasioned them. On the other hand, stadia built for mega-events often continue to be a financial burden for hosts after the event (Alm et al., 2016), so one might argue the opposite and claim that the actual costs exceed the construction cost Third, we only consider direct revenues and direct costs, acknowledging that results might be different if one took into account indirect revenues and indirect costs. Fourth and last, hosting a mega-event can have other reasons than purely economic ones, ranging from political posturing (Grix, 2013) to entrepreneurial urban development (Lauermann, 2014; Trubina, 2019). An economic evaluation such as this one can only shed light on one element of a fuller cost–benefit analysis.

### Analysis

#### Revenues

**Figure 2.**
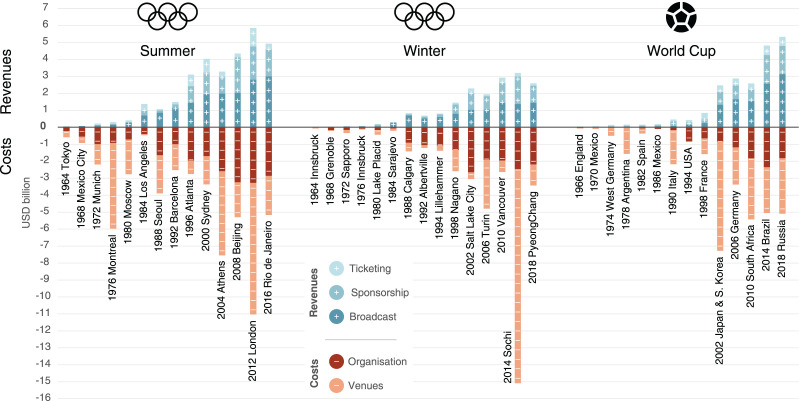
Comparison of revenues and costs of the Olympic Games and the Football World Cups, 1964–2018. Notes: The following data are missing: Ticketing revenue for 1968 Mexico City; sponsorship revenue for 1968 Mexico City, 1964 and 1976 Innsbruck, 1972 Sapporo and 1966 England; cost of venues for 1986 Mexico). For Rio 2016 venue costs rely on the latest estimate, as final costs were not published by early 2022. For PyeongChang 2018 international sponsorship has been estimated based on previous editions, as the IOC had not yet published figures in early 2022. USD values for Sarajevo 1984 need to be taken with caution, as the Yugoslavian dinar went through hyperinflation during the period of Games preparation.

Figure 2 shows the longitudinal evolution of revenues and costs of the Summer and Winter Olympic Games and the Football World Cup. The Summer Games in Los Angeles in 1984 were the first to pass the mark of USD_2018_ 1 billion in revenues, then Nagano in 1998 for the Winter Games, and finally the World Cup in Japan/South Korea in 2002. The time to pass the USD_2018_ 2 billion mark was much shorter: this happened after 12 years for the Summer Games (Atlanta 1996, which, in fact, skipped to just over USD_2018_ 3 billion) and 4 years for the Winter Games (Salt Lake City 2002), whereas for the World Cup the 2002 edition had jumped the USD_2018_ 1 billion and the USD_2018_ 2 billion mark at once to bring in almost USD_2018_ 2.3 billion, up from just USD_2018_ 0.9 billion in the previous edition in France in 1998.

At the end of the 2010s, the revenues of the Summer Games and the World Cup were of roughly similar size and fluctuated at around USD_2018_ 5 billion, whereas those of the Winter Olympics hovered at around USD_2018_ 3 billion. It is interesting to note that the World Cup staged a catch-up race with the Summer Olympics in the 2010s, as the editions in Brazil in 2014 and in Russia in 2018 allowed a jump in revenues, driven by sponsorship and broadcasting, from about USD_2018_ 2.5 billion for the World Cup 2010 in South Africa to approximately USD_2018_ 5 billion at the end of the 2010s.

Calculated over the last three editions of each event to smooth out fluctuations, the Summer Olympics hold a slight lead over the World Cup in total revenues (USD_2018_ 5 billion in revenue to the World Cup’s USD_2018_ 4.2 billion), whereas the Winter Olympics come in at USD_2018_ 2.9 billion (see Table 2). Broadcast revenue accounts for about 50% of total revenues in all three events (slightly more for the World Cup), sponsorship for another 30% to 35% and ticketing revenue for about 10% to 15%, as Table 2 indicates. While broadcasting has always made up the lion’s share of revenues, the weight of sponsoring has risen over time, with a notable increase since the 1990s. In the Beijing 2008 and Sochi 2014 Olympics, sponsorship revenue even exceeded broadcasting revenue slightly (Figure 2).

**Table 2.** Total and mean values for revenues, costs, profit/loss and return on investment for the Summer and Winter Olympic games and the Football World Cup, 1964–2018 (all monetary values in USD_2018_ million; the volatility index indicates higher volatility with increasing distance from zero).

	Summer Olympic Games				Winter Olympic Games				World Cup				
	Total	Mean	Volatility	Mean last three	Total	Mean	Volatility	Mean last three	Total	Mean	Volatility	Mean last three	Total
**Revenues**	**30,413**	**2339**	**0.86**	**5033**	**17,213**	**1434**	**0.78**	**2902**	**20,441**	**1572**	**1.18**	**4240**	**68,066**
Broadcast revenue	16,507	1179	0.88	2613	9388	626	0.87	1426	10,898	778	1.37	2414	36,793
Sponsorship revenue	9603	739	1.06	1876	6587	549	1.00	1260	6408	493	1.21	1344	22,597
Ticketing revenue	4375	337	0.96	544	1393	93	1.01	216	3174	227	0.87	482	8942
**Costs**	**54,510**	**3894**	**0.75**	**7164**	**37,023**	**2468**	**1.53**	**7047**	**33,438**	**2572**	**0.94**	**5192**	**124,972**
Organising costs	22,107	1579	0.67	3130	16,254	1084	0.88	2207	10,008	715	1.12	2004	48,368
Venue costs	32,403	2315	1.15	4035	20,769	1385	2.31	4840	23,524	1810	1.03	3188	76,696
**Profit/loss**	**−23,175**	**−1783**	**−1.22**	**−2131**	**−19,255**	**−1605**	**−2.08**	**−4145**	**−13,111**	**−1093**	**−1.33**	**−952**	**−55,540**
**ROI**	**−43%**	**−29%**		**−23%**	**−52%**	**−37%**		**−31%**	**−39%**	**−47%**		**−18%**	**−44%**

The volatility index in Table 2 reveals the highest volatility in revenues for the World Cups, mostly due to the large jumps in broadcasting revenue. Sponsorship is the most volatile revenue source for the Olympic Games, due to its large variation between host cities.

#### Costs

Costs also follow a general upward trend. The Summer Games of our sample have always cost more than USD_2018_ 1 billion since Munich in 1972, with the exception of Los Angeles in 1984. The low cost of Los Angeles 1984 (just below USD_2018_ 0.5 billion) is noteworthy, as it is even lower than that of Tokyo 1964 20 years earlier (USD_2018_ 0.6 billion). It is useful to know that Los Angeles was the only bidder for the 1984 Olympics at the time and was able to negotiate down infrastructural requirements for the Olympic Games, resulting in the significant use of existing infrastructure (Burbank et al., 2001). More recent Summer Olympics have seen total costs (organisation and venues combined) range between USD_2018_ 5 billion and USD_2018_ 11 billion (the latter being a record that goes to London 2012).

The cost of the Winter Olympics rose to above USD_2018_ 1 billion with the Calgary Games of 1988 and to more than USD_2018_ 2 billion with Nagano 1998, barely ten years later. It has since never been below that threshold, and once went up as high as USD_2018_ 15 billion for the 2014 Sochi Olympics. All of the venues in Sochi were built from scratch and a significant degree of corruption likely also drove up costs (Müller 2014).

For the World Cup, Italy 1990 is an early expensive event at USD_2018_ 2.1 billion, but the following World Cup in the United States in 1994 brought costs down to USD_2018_ 0.8 billion in a no-frills exercise similar to LA 1984. World Cup costs hit an all-time high for the World Cup in Japan/South Korea with USD_2018_ 7.3 billion, and have never fallen below USD_2018_ 3 billion since.

When looking at the three most recent editions of each event (Table 2), the Summer and Winter Olympics have had about similar costs of around USD_2018_ 7 billion each. The enormous costs of Sochi 2014 drive up the mean for the Winter Olympics, which would hover more around USD_2018_ 3.5 billion, if Sochi was excluded. The average costs of the last three World Cups ran to about USD_2018_ 5 billion, so about two-thirds of the costs of the Olympics. For the whole sample, costs of organisation make up about 30% to 40% of total costs, whereas venue costs run to 60% to 70% of total costs.

The volatility index shows that venue costs are much more volatile than costs of organisation for the Olympic Games, while volatility is about equal for both cost types for the World Cup. The extremely strong volatility of venue costs for the Winter Olympic Games is noteworthy, even when accounting for the extreme event of Sochi 2014.

#### Revenues and costs relative to size

**Figure 3.**
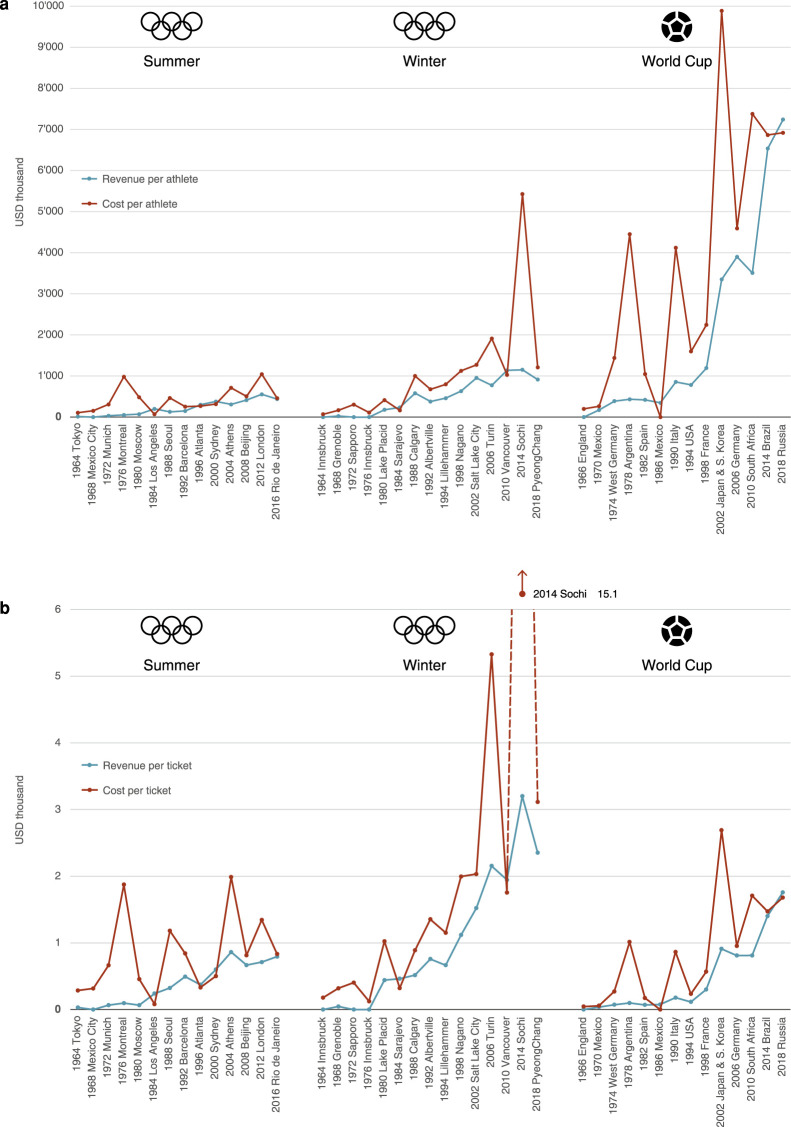
(a) and (b) Revenues and costs of the Olympic Games and the Football World Cup in relation to size, 1964–2018 (a) per athlete, (b) per ticket (all values in USD_2018_). Notes: See Figure 2.

As the size of the Olympic Games and the World Cup has also increased over time, we examine the development of revenues and costs relative to two indicators of size: the number of athletes (Figure 3(a)) and the number of tickets (Figure 3(b)). The positive slope in both graphs shows that revenues and costs have grown proportionately more than the size of the event, as measured by athletes and tickets. The costs per athlete are about two to three times as high for the Winter Olympics as for the Summer Olympics, but the same is true for the revenues per athlete. The World Cup has the highest costs and revenues per athlete, at between USD_2018_ 6 and 8 million per athlete in the most recent events. The Olympics are much cheaper, at around USD_2018_ 0.5 million of revenue and costs per athlete for the Summer Games and around USD_2018_ 1 to 2 million of costs and USD_2018_ 1 million of revenue per athlete for the Winter Games.

Revenues and costs per ticket have also risen over time. They are the highest for the Winter Olympics, reaching some USD_2018_ 2000 to 3000 of revenue per ticket over the last few Winter Games, with costs per ticket ranging from USD_2018_ 2000 to 15,000. For the World Cups, both revenues and costs have clocked in at around USD_2018_ 1500 per ticket in the most recent events. Compared to the World Cups and the Winter Olympics, the Summer Olympics are cheap to host, using the number of tickets as a yardstick. Costs per ticket have fluctuated between USD_2018_ 800 and 1400 for recent events and reached an all-time high for Athens 2004 at just above USD_2018_ 2000. Revenues are somewhat lower, at between USD_2018_ 600 and 800 per ticket.

These figures show that the Winter Olympic Games are the most expensive to organise in relative terms, but also bring in the highest revenue. By contrast, if one wanted to attract the largest number of spectators (for example to maximise tourist spending) at the lowest relative cost, hosts would have to opt for the Summer Games. But does the revenue cover the costs in any of these events? In other words, are these events at all profitable?

#### Surplus/deficit and return on investment

**Figure 4.**
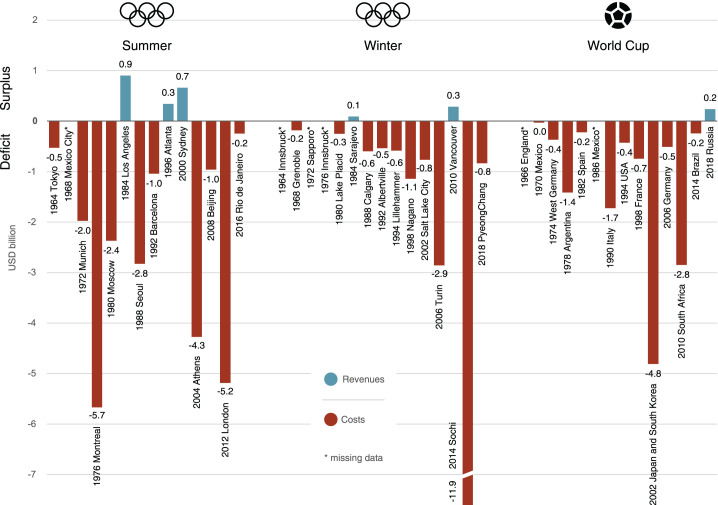
Surplus/deficit of Olympic Games and World Cups, 1964–2018 (all values in USD_2018_). Notes: See Figure 2.

The comparison of costs against revenues in Figure 4 shows that the large majority of events in our sample ran a deficit. The Sochi 2014 Olympics posted the highest deficit of all 43 events at close to USD_2018_ 12 billion. Leaving aside this exceptional case, deficits have reached up to almost USD_2018_ 6 billion for the Summer Olympics (Montréal, 1976), close to USD_2018_ 3 billion for the Winter Olympics (Turin, 2006) and almost USD_2018_ 5 billion for the World Cup (Japan/South Korea 2002). The Summer Olympic Games have had editions with deficits of USD_2018_ 2 billion and more since the 1970s, whereas such large deficits have only become common with the Winter Olympics and the World Cup later, around the 2000s.

It is possible for these events, however, to be profitable. Los Angeles 1984, Atlanta 1996 and Sydney 2000 were profitable Summer Games, Vancouver 2010 was a profitable Winter Games, and Russia 2018 was a profitable World Cup (the profit for Sarajevo 1984 needs to be taken with caution, see notes in Figure 2). Profitable events in our sample show a conjuncture of lower-than-average costs and higher-than-average revenues; just one or the other alone does not appear to be sufficient for profitability. In general, however, any profits were significantly lower than the losses in other events.

**Figure 5.**
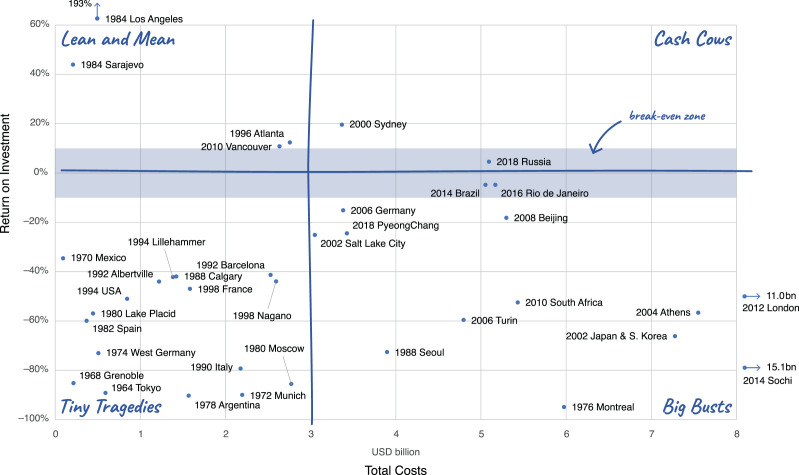
The financial performance of Olympic Games and World Cups, 1964–2018: return on investment (surplus/deficit divided by costs) plotted against total costs (all values in USD_2018_). Notes: See Figure 2.

Figure 5 considers the deficit/surplus in relation to the total cost This measure – known as return on investment (ROI) – is important, since small absolute surpluses can be relatively large if compared to total costs and the other way round. The quadrant of ‘lean and mean’ events includes profitable events with low costs. It is there we find Los Angeles 1984 (with a record ROI of close to 200%), Sarajevo 1984, Atlanta 1996 and Vancouver 2010. There are very few ‘cash cows’, that is, expensive but profitable events: Sydney 2000 is the only to make it into that zone. The Olympics in Rio in 2016 (venue costs are provisional) and the World Cups in Brazil in 2014 and in Russia in 2018 are situated in a ‘break-even zone’ that covers the corridor of ROI ± 10%.

Below the horizontal axis in Figure 5, we find all editions of our sample that ran a deficit. Until about the end of the 1980s, most of those had total costs below USD_2018_ 3 billion, which made them what we qualified as ‘tiny tragedies’. From the 2000s onwards, these events have become ‘big busts’, as costs kept rising but ROIs did not improve. Some of the most expensive events have very low ROIs (e.g. Sochi, 2014 at − 79%, Japan/South Korea 2002 at − 66%, London 2012 at − 47%), indicating that high costs are often not offset by high revenues. Indeed, not a single event with costs above ca. USD_2018_ 5 billion reported a positive ROI. Overall, however, as the pattern of points in the scatter plot suggests, there is no significant correlation between costs and ROI, as also low-costs events such as Tokyo 1964 and Grenoble 1968 had dismal returns on investment.

Examining the entire time period of our sample, an Olympic Games, Summer or Winter, incurred an average loss of about USD_2018_ 1.5 billion, whereas the World Cup incurred an average loss of about USD_2018_ 1 billion. The mean ROIs across all events in our sample are thus negative for all three event types: the Summer Games are the least bad at − 25% mean ROI, followed by the Winter Games ( − 37%) and the World Cup ( − 47%). However, the mean ROI of the last three events of each event type post much better results: − 9% for the Summer Olympics, − 31% for the Winter Olympics and − 18% for the World Cup. There is, then, cautious hope that the structural deficit might be decreasing.

## Conclusion

The Games can no more have a deficit than a man can have a baby.

Jean Drapeau, Mayor of Montréal, 1973 (CBC, 1999)

Jean Drapeau’s infamous quip had been proven wrong for the Olympics in Montréal. This paper has been the first to conduct a systematic, long-term study that proves him wrong for indeed the majority of the Olympics and the World Cups: most mega-events are not profitable. Out of 36 events for which we have robust data, 31 events, or 86%, ran a financial deficit. The Summer Olympics have the smallest proportion of events with a deficit: 10 out of 13, equivalent to 77%. For the World Cup, by contrast, all events but one (the World Cup 2018 in Russia) posted a deficit, corresponding to 92% of all World Cups. As we have adopted a conservative approach for calculating costs, these percentages should be understood as lower bounds.

In other words, these events suffer from what can be called a ‘structural deficit’, that is, a deficit that is persistent and systematic, and therefore not an outcome of poor individual decision-making or specific hosting conditions. The finding of a systematic presence of deficits, what we call ‘structural deficits’, is important for two reasons. On a theoretical level, it shows that deficits are persistent and therefore not an outcome of poor individual decision-making or specific adverse conditions for hosting the event, such as economic downturns, high corruption, protests or others. Our findings, therefore, lend support to theoretical explanations that predict a systematic presence of underestimated costs and overestimated benefits for mega-events. We are, however, not able to establish the individual contribution of each of the different explanations to the final outcome nor to trace the exact mechanisms for each case.

On the economic level, the results show that the World Cup and the Olympic Games are not financially viable in and of themselves. In other words, the IOC and FIFA would long have gone bankrupt, if they had to shoulder the direct costs of their events from the revenues these events create. If these events still continue today, this is because they receive subsidies external to the event itself, mostly for venue construction. In theory, these subsidies could come from private sources, for example from clubs or investors planning to operate stadiums profitably. Research indicates, however, that these are often public subsidies, as many stadiums cannot operate at a profit after the event (Alm et al., 2016).

Our analysis then demonstrates that the supposed prize on offer when bidding for these events in fact has a negative financial value in most cases. These events, in their current form of organisation, are unable to pay their own way and would stop without external subsidies. If they still create sizable profits for the IOC and FIFA, this is because these governing bodies have secured authority over the most important revenue streams over time, while remaining liable for only a small part of the costs. Our findings, however, urge a reconsideration of the bargaining position of IOC and FIFA in the awarding of events, as they are not offering the rights to a profit-making business deal but asking for subsidies for a loss-making venture.

For cities and countries, our study provides a systematic source of information for two of the major types of costs they will incur: organisation cost and venue cost. It also demonstrates that low venue costs were essential for an event to make a surplus. Thus, among the six events that were profitable, all but one (Russia 2018) had venue costs that were much below the long-time average for the event. It is therefore paramount for future hosts to reuse existing venues to the greatest extent possible, if they want to reduce the financial risk associated with this event. Finally, if cities aim to primarily attract visitors by hosting these events, we have shown that the Summer Games and the World Cup are more cost-efficient per ticket than the Winter Games.

On a more positive note, the return-on-investment for these events has become less worse over recent editions. Together with an emphasis on cutting costs in the Olympics (IOC 2014), this gives cautious reason for the hope that mega-events might eventually come closer to at least breaking even. For host cities and countries to participate in such a surplus, however, they need to lobby for more equitable sharing of revenues and risks. While IOC and FIFA may insist on fair play on the pitch, they do not seem to mind that in the current game of mega-events the cards are mightily stacked in favour of them.

For a better understanding of the drivers behind revenues, costs and profit/loss regression analyses to identify predictors would be a useful next step. Are certain political and economic systems more likely to produce high costs and high deficits than others? (We note that four of the five profitable events in our sample took place in North America and Australia.) Do the wealth of a host country and city, or the size of a city play a role? Does the level of infrastructural development of the host have an influence on costs? Do corruption and transparency influence the outcomes? As current theoretical explanations do not consider the economic, social and political context of event hosts, answering these questions would help us arrive at a more nuanced conceptual understanding of the circumstances under which financial profit and loss occur and how to better manage them.

## Data availability

The dataset for this article is available at (Müller et al., 2022).
